# Myofasziitis unter Nivolumab-Therapie

**DOI:** 10.1007/s00393-021-01001-7

**Published:** 2021-04-22

**Authors:** M. Krusche, U. Schneider, C. Geisler, S. Keller, W. Stenzel, S. Ohrndorf

**Affiliations:** 1 Medizinische Klinik mit Schwerpunkt Rheumatologie und Klinische Immunologie, Charité – Universitätsmedizin, Charitéplatz 1, 10117 Berlin, Deutschland; 2 Klinik für Dermatologie, Venerologie und Allergologie, Charité – Universitätsmedizin, Charitéplatz 1, 10117 Berlin, Deutschland; 3 Klinik für Radiologie, Charité – Universitätsmedizin, Charitéplatz 1, 10117 Berlin, Deutschland; 4 Institut für Neuropathologie, Charité – Universitätsmedizin Berlin, Charitéplatz 1, 10117 Berlin, Deutschland

**Keywords:** Checkpointinhibitoren, IrAEs, Myalgie-Kontraktur-Myositis, Fasziitis, Eosinophilie, Checkpoint inhibitors, IrAEs, Myalgia contraction myositis, Fasciitis, Eosinophilia

## Abstract

Wir schildern den Fall einer 73-jährigen Patientin mit malignem Melanom, die eine rasch progrediente Dermatosklerose der Arme und Beine sowie Myalgien und Beugekontrakturen unter der Therapie mit dem Immuncheckpoint-Inhibitor Nivolumab entwickelte. Bildmorphologisch und bioptisch konnte die Diagnose einer Myofasziitis gesichert werden. Nach Rücksprache mit den behandelnden Dermatoonkologen wurde die Nivolumab-Therapie bei gutem Ansprechen des Malignoms pausiert und eine immunmodulierende Therapie mit Methotrexat und Prednisolon eingeleitet. Immuncheckpointinhibitoren können vielfältige immunvermittelte Nebenwirkungen induzieren und auch rheumatologische Krankheitsbilder imitieren. Das Auftreten einer Myofasziitis unter Immuncheckpointinhibition ist nur in wenigen Fällen in der Literatur berichtet. Sinnvoll für die Diagnostik sind insbesondere die Bestimmung der eosinophilen Leukozyten, eine bildgebende Diagnostik mittels Sonographie und/oder MRT sowie die Gewinnung einer Gewebebiopsie. Die weitere onkologische und rheumatologische Therapiesteuerung sollte in enger interdisziplinärer Abstimmung erfolgen.

## Abstract (en)

We report the case of a 73-year-old female patient with malignant melanoma who developed rapidly progressive dermatosclerosis of the arms and legs as well as myalgia and flexion contractures during treatment with the immune checkpoint inhibitor nivolumab. The diagnosis of a myofasciitis was confirmed by imaging and biopsy. Following consultation with the treating dermato-oncologists nivolumab treatment was paused and treatment with methotrexate and prednisolone was initiated. Immune checkpoint inhibitors can induce a variety of immune-mediated side effects and can also imitate symptoms of rheumatological diseases. The occurrence of myofasciitis under immune checkpoint inhibition has been reported in the literature only in a few cases. Further oncological and rheumatological treatment management should be carried out in close interdisciplinary coordination.

## Anamnese

Wir berichten über den Fall einer 73-jährigen Patientin, welche im Dezember 2020 mit der klinischen Verdachtsdiagnose einer eosinophilen Fasziitis von einem niedergelassenen Rheumatologen stationär eingewiesen wurde.

Die Patientin gab bei Aufnahme an, dass sie eine Hautverhärtung der Unter- und Oberschenkel sowie der Unter- und Oberarme verspüre, die sich seit ca. 3 Monaten progredient entwickelt hätte. Weiterhin klagte die Patientin über diffuse Schmerzen in der gesamten Extremitätenmuskulatur. Aufgrund der ausgeprägten Hautverdickung und der muskulären Schmerzen sei das vollständige Strecken der Beine und der Arme nicht mehr möglich, was sowohl das Laufen als auch das Ankleiden stark behindern würde.

**Figure 1:**
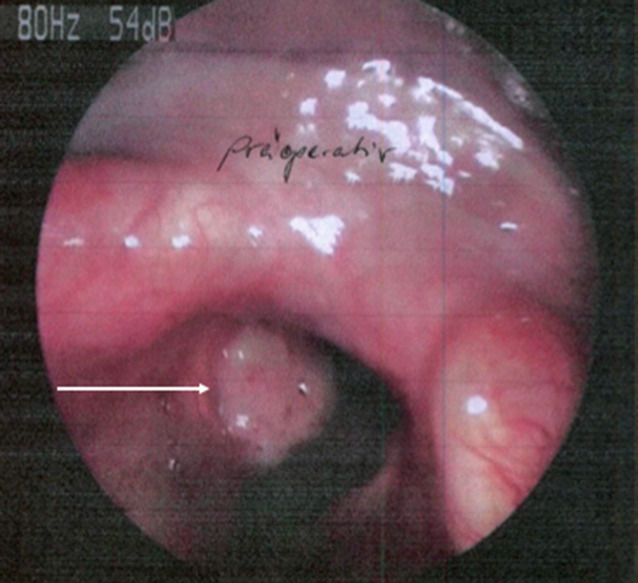

Bei der Patientin waren erstmalig 2011 ein malignes Melanom am Unterschenkel sowie ein Zweitmelanom am Oberschenkel diagnostiziert worden, welche beide erfolgreich operativ entfernt worden waren. Im Jahr 2019 wurde bei der Patientin dann ein malignes Melanom Stadium 4 (R1) beider Stimmlippen festgestellt (Abb. 1). Da die Patientin eine operative Kehlkopfentfernung abgelehnt hatte, wurde aufgrund des Alters eine palliative Monotherapie mit dem Checkpointinhibitor Nivolumab im Juni 2019 eingeleitet, welche bis September 2020 fortgeführt wurde. Aufgrund ausgeprägter Durchfälle mit Hyponatriämie sowie Arthralgien beider Ellenbogen‑, Hand- und Kniegelenke wurde Nivolumab im September bei Verdacht einer Checkpointinhibitor-induzierten Kolitis pausiert. An weiteren Vorerkrankungen waren eine COPD sowie eine arterielle Hypertonie bekannt.

## Befund

**Figure 2:**
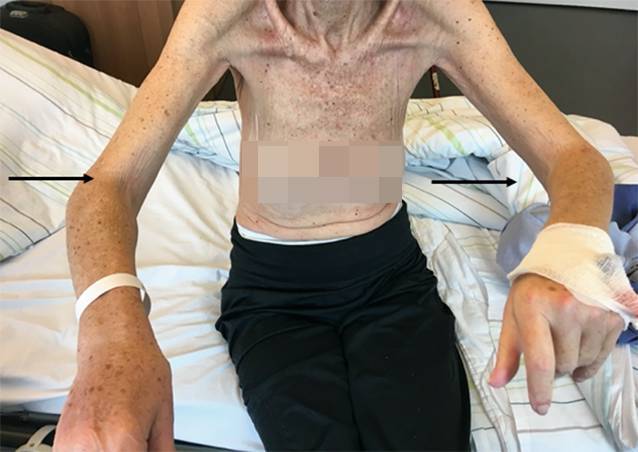

In der körperlichen Untersuchung bei Aufnahme zeigte sich eine deutliche Dermatosklerose der Arme und Beine („modified Rodnan skin score“: 18) mit ausgeprägten Beugekontrakturen der Ellenbogengelenke (Abb. 2) sowie diskreten Beugekontrakturen der Kniegelenke. Die Kraftgradprüfung war aufgrund der Kontrakturen nur eingeschränkt möglich. Paresen oder Arthritiszeichen konnten in der körperlichen Untersuchung nicht detektiert werden. Laborchemisch sah man bis auf ein minimal erhöhtes CRP von 7,1 mg/dl (normal < 5,0 mg/dl) keine Auffälligkeiten, insbesondere Kreatinkinase (CK), Anteil der Eosinophilen im Differenzialblutbild sowie Laktatdehydrogenase (LDH) waren normal. In der Autoimmunserologie waren der ANA-Titer mit 1:320 (nukleolär, fein gesprenkelt) und in der ENA-Differenzierung die Ro‑/La-Antikörper positiv. Die Myositis-spezifischen Antikörper waren negativ. Weitere laborchemische oder klinisch-anamnestische Hinweise für das Vorliegen eines Sjögren-Syndroms lagen nicht vor.

**Figure 3:**
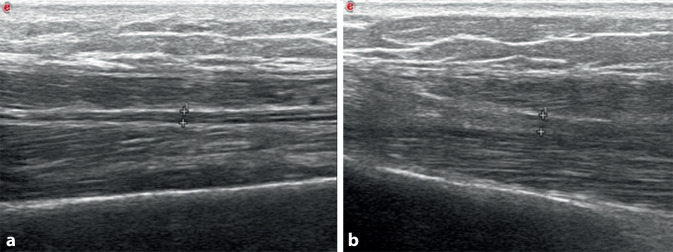

**Figure 4:**
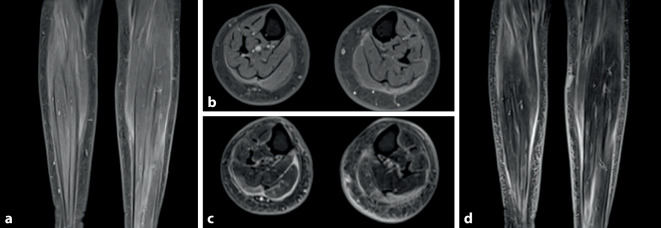

Zur weiterführenden Diagnostik ergänzten wir eine Sonographie der Muskulatur und Faszien. Hier zeigte sich insbesondere im rechten M. quadriceps femoris und M. gastrocnemius lateral wie auch in sonstigen untersuchten Muskeln bzw. -gruppen eine deutliche Verbreiterung der Muskelfaszien (Abb. 3) mit sonographischen Hinweisen für eine begleitende Myositis. Zur weiteren Einordnung erfolgte eine MRT beider Unterschenkel, die eine ödematöse Verbreiterung der Faszien beider Mm. gastrocnemii mit geringen myositischen Veränderungen ergab (Abb. 4).

**Figure 5:**
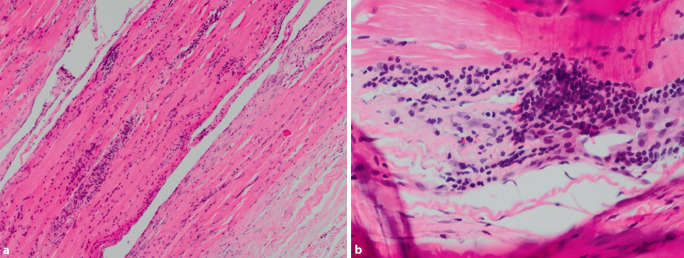

In der tiefen Haut‑/Muskelbiopsie zeigte sich ein T‑Zell-dominiertes Entzündungszellinfiltrat, welches sowohl endo-, peri- als auch epimysial (im Bereich der Faszie) lokalisiert war (Abb. 5). Die kardiologische Diagnostik mittels EKG und Echokardiographie zum Ausschluss einer kardialen Mitbeteiligung war unauffällig. In Zusammenschau der Befunde konnte die Diagnose einer Nivolumab-induzierten Myofasziitis gestellt werden.

## Therapie und Verlauf

Da sich in der HNO-ärztlichen Kontrolle sowie im Verlaufs-CT des Halses kein Hinweis auf ein Rezidiv des Stimmlippenmelanoms zeigte, wurde die Therapie mit dem Checkpointinhibitor dauerhaft pausiert und eine Therapie mit Methotrexat s.c. 15 mg pro Woche und Prednisolon mit initial 60 mg p.o. pro Tag (in absteigender Dosierung) eingeleitet.

## Diskussion

Wir schildern den Fall einer 73-jährigen Patientin mit metastasiertem Melanom, die eine Nivolumab-induzierte Myofasziitis in der Muskulatur der oberen und unteren Extremitäten entwickelte.

Weltweit erkranken fast 200.000 Menschen jährlich an einem malignen Melanom [1]. Für Frauen liegt das mittlere Erkrankungsalter bei 60 und für Männer bei 64 Jahren.

Als adjuvante Therapie beim fortgeschrittenen malignen Melanom (im Stadium III oder IV) kommen unter anderem Immuncheckpointinhibitoren zum Einsatz, wie z. B. die beiden „Programmed cell death protein 1“(PD-1)-Antikörper Nivolumab und Pembrolizumab [2].

Bekanntermaßen wird unter Immuncheckpointinhibition (ICI) bei Tumorerkrankungen eine Vielzahl immunvermittelter Nebenwirkungen (irAE) beschrieben. Hierbei können fast alle Organsysteme betroffen sein [3]. Insbesondere für die Rheumatologie relevant sind die rheumatologischen immunvermittelten Nebenwirkungen („rheumatic immune-related adverse events“ [rh-irAE]) [4]. In einer Übersichtsarbeit von Versapohl et al. wird die Prävalenz der rh-irAE zwischen 2,3 und 6,6 % angegeben. Am häufigsten kommt es zu Arthralgien, Arthritiden und Myositiden. Das Auftreten von Vaskulitiden, Sarkoidosen oder Kollagenosen wird seltener beschrieben.

Unspezifische Myalgien werden in ca. 1–4 % der Patienten als irAE berichtet [5, 6]. Darüber hinaus können Immuncheckpointinhibitoren auch eine Myositis auslösen. So identifizierten Sato et al. in einer retrospektiven Studie 127 Myositisfälle (1,7 %) bei insgesamt 7604 Patienten mit ICI-Therapie [7]. Insbesondere Nivolumab scheint vermehrt mit dem Auftreten von ICI-vermittelten Myositiden assoziiert zu sein [8, 9]. In der Arbeit von Toat et al. konnte weiterhin gezeigt werden, dass in den meisten Fällen einer ICI-induzierter-Myositis zwar eine deutliche Erhöhung der CK nachweisbar war, aber sowohl die Myositis-assoziierten Antikörper als auch die ANA zumeist negativ waren [10]. In Fällen einer schweren Myositis (bulbäre Symptomatik, Dysphagie, Dyspnoe, Myokarditis) sollte die ICI-Therapie pausiert werden. In diesen Fällen kamen u. a. hoch dosierte Glukokortikoide sowie intravenöse Immunglobuline und/oder Plasmapheresen zum Einsatz [11].

Neben der Muskulatur können unter ICI-Therapie auch Veränderungen der Faszien auftreten. Insbesondere das Vorkommen einer eosinophilen Fasziitis wird häufiger beschrieben. In einem Review von Chan et al. identifizierten die Autoren 15 Patientenfälle, die unter ICI-Therapie eine eosinophile Fasziitis entwickelten [12]. Hiervon waren 1 Patient mit einer Kombinationstherapie aus Nivolumab und Ipilimumab und 5 Patienten mit einer Nivolumab-Monotherapie behandelt worden. Der Zeitraum des Auftretens der klinischen Symptomatik nach ICI-Therapieeinleitung war variabel (1,5 bis 24 Monate). Interessanterweise sollen erhöhte Eosinophilenwerte im Blut zum Beginn der ICI-Therapie mit einem besseren Therapieansprechen und Überleben bei Melanompatienten, aber auch mit vermehrten Nebenwirkungen assoziiert sein [13].

Nicht immer sind die Veränderungen der Muskelfaszie jedoch durch Eosinophile vermittelt. So wird neben der eosinophilen Fasziitis auch das Auftreten eines „eosinophilic fasciitis-like“ bzw. „scleroderma-like syndrome“ unter ICI-Therapie berichtet [14]. In der Publikation von Rischin et al. wird der Fall eines 55-jährigen Patienten mit metastasiertem Melanom beschrieben, der unter Nivolumab-Therapie eine rein lymphozytäre Fasziitis der Unterarme entwickelte [15].

Neben einer isolierten Faszienreaktion unter ICI-Therapie ist auch (analog zu unserem Fall) eine Mitbeteiligung der Muskulatur im Sinne einer Myofasziitis beschrieben [14, 16].

Klinisch scheint es hier in Abgrenzung zur eosinophilen Fasziitis nicht zum Auftreten einer Peau d’orange oder des Groove-Signs zu kommen [14].

Therapeutisch werden zumeist Glukokortikoide eingesetzt. Weiterhin wurden vermehrt auch Methotrexat und vereinzelt intravenöse Immunglobuline angewendet [12, 14]. In Ausnahmefällen wurde hier die ICI-Therapie pausiert/abgesetzt.

Besonders wichtig ist, dass die Behandlung von Patienten mit rh-irAE in interdisziplinären Teams mit Rheumatologen und Onkologen erfolgen sollte [17]. In kritischen Fällen sollte das Pausieren/Absetzen der ICI-Therapie in gegenseitiger Absprache in Erwägung gezogen werden [10].

Differenzialdiagnostisch sollte bei dem Auftreten einer Fasziitis auch an das Vorliegen einer paraneoplastischen Genese gedacht werden. Aufgrund des zeitlichen Verlaufes und des guten Tumoransprechens gehen wir jedoch in dem geschilderten Fall von einer Nivolumab-induzierten Myofasziitis aus.

## Fazit für die Praxis

- Unter Checkpointinhibition kann in seltenen Fällen eine (Myo‑)Fasziitis auftreten.
- Diagnostisch sind insbesondere die Bestimmung der Eosinophilen sowie weiterführende bildgebende Diagnostik mittels Sonographie/MRT und eine Gewebebiopsie sinnvoll.
- Die weitere onkologische und rheumatologische Therapiesteuerung sollte in einer engen interdisziplinären Abstimmung erfolgen.
